# Dynamic Lissajous patterns for real time identification and localization of power quality disturbance

**DOI:** 10.1038/s41598-025-10218-4

**Published:** 2025-09-05

**Authors:** Pampa Sinha, Snehalika Snehalika, Ranjith Kumar Gatla, P. Shashavali, Devineni Gireesh Kumar, Idamakanti Kasireddy, D. S. Naga Malleswara Rao, Hassan Abdurrahman Shuaibu, Taha Selim Ustun

**Affiliations:** 1 School of Electrical Engineering, KIIT, Bhubaneswar, 751024 India; 2 Department of Computer Science and Engineering (Data Science), Institute of Aeronautical Engineering, Hyderabad, Telangana 500043 India; 3 Department of EEE, S K University College of Engineering & Technology, Ananthapur, Andhra Pradesh 515003 India; 4 Department of EEE, B V Raju Institute of Technology, Narsapur, Telangana 500092 India; 5 EEE Department, Vishnu Institute of Technology, Bhimavaram, 534202 India; 6 Department of EEE, Gokaraju Rangaraju Institute of Engineering and Technology, Hyderabad, 500090 India; 7 Department of Electrical, Telecommunications and Computer Engineering, Kampala International University, Kampala, Uganda; 8 Fukushima Renewable Energy Institute, Koriyama, 963-0298 Japan

**Keywords:** Dynamic lissajous patterns, Real-Time identification, Power quality disturbances, Localization, Microgrids, Voltage signals, Current signals, Adaptive thresholds, Event detection, Disturbance localization, Grid stability, Power quality monitoring, Electrical and electronic engineering, Energy infrastructure

## Abstract

This study proposes a novel and computationally efficient method for real-time identification and localization of power quality (PQ) disturbances in microgrids using dynamic Lissajous patterns formed by voltage and current waveforms. Each power disturbance—such as sag, swell, harmonic distortion, and transients—induces a unique geometric deformation in the Lissajous figure, which serves as a visual signature of the event. Key geometric and statistical features, including area, skewness, kurtosis, and centroid deviation, are extracted from these dynamic patterns to construct robust indices for classification. Adaptive thresholds for each feature are determined dynamically, enabling accurate event detection without prior knowledge of the microgrid configuration. Numerical analysis demonstrates that the proposed method achieves high precision in detecting and distinguishing PQ disturbances, even under overlapping or noisy conditions. For instance, the method records Euclidean distance values of 0.7071 for swell and 0.5657 for harmonic distortion, while maintaining zero deviation for pure signals, validating its classification accuracy. The system exhibits strong resilience to unstable grid conditions and is computationally lightweight, making it suitable for real-time deployment in embedded devices within microgrids. Comparative evaluation reveals superior performance in terms of speed, accuracy, and false positive minimization, highlighting the potential of dynamic Lissajous patterns as a powerful tool for advanced PQ monitoring in smart grid infrastructures.

## Introduction

Power quality (PQ) monitoring has become increasingly critical due to the proliferation of distributed energy resources (DERs), nonlinear loads, and bidirectional power flow in smart grids and microgrids. Traditional techniques such as Fast Fourier Transform (FFT), Wavelet Transform (WT), and Total Harmonic Distortion (THD) analysis are commonly used for detecting disturbances like voltage sags, swells, harmonics, and transients. However, these methods suffer from several limitations in dynamic grid environments: they often fail to detect overlapping or transient events, require extensive parameter tuning (e.g., mother wavelet selection), are computationally intensive, and may not operate in real time. Moreover, machine learning-based approaches demand large labelled datasets and struggle to generalize across varying disturbance profiles, especially in microgrids where operating conditions change frequently. The motivation is further driven by the need for advanced techniques capable of not only detecting power quality disturbances but also pinpointing their exact location without relying on complex, prior network configurations. Current methodologies often fall short in dynamic and rapidly changing environments, making it difficult to ensure the optimal performance of microgrids. By leveraging the dynamic characteristics of Lissajous patterns formed by voltage and current signals, this study presents a novel solution that addresses these limitations. The distinct geometric features of Lissajous patterns, such as area, skewness, and kurtosis, provide an intuitive and effective means to differentiate between various types of power quality disturbances. This approach enables rapid identification and precise localization of disturbances, offering significant improvements in monitoring efficiency, system resilience, and operational reliability. This research aims to bridge the gap between traditional power quality monitoring methods and the growing demand for real-time, adaptable, and robust solutions that enhance the stability and performance of microgrids in increasingly complex environments.

The core research problem addressed in this study is the lack of a real-time, accurate, and computationally efficient method for identifying and classifying power quality disturbances in dynamic and resource-constrained environments. Current methods either compromise on speed or accuracy, or are unsuitable for embedded deployment due to their complexity. To overcome these challenges, this work proposes a novel approach that utilizes geometric features derived from dynamic Lissajous patterns—parametric plots of voltage versus current—to reliably capture the signature of PQ disturbances. By focusing on low-complexity, statistically rich features such as area, skewness, and kurtosis, the proposed method enables real-time detection and classification without the need for transformations or training data. This research addresses the urgent need for scalable, low-latency PQ monitoring systems that are resilient under diverse operating conditions, including overlapping events, grid instability, and noise.

### Literature survey

Power quality disturbances in microgrids, such as voltage sags, swells, harmonics, and transient faults, pose significant challenges for their stable operation and effective integration with the main grid. Traditional power quality monitoring (PQM) methods often struggle to detect disturbances in real-time, particularly in complex systems with overlapping disturbances or unstable grid conditions. To address these challenges, the use of dynamic Lissajous patterns, derived from voltage and current signals, has emerged as an innovative and highly effective technique for the real-time identification and localization of power quality events.

#### Lissajous figures for power quality disturbance detection

Lissajous figures are graphical representations of the relationship between two periodic signals, typically voltage and current waveforms. When applied to power systems, Lissajous patterns exhibit distinct shapes under different operating conditions, including during disturbances such as voltage sags, swells, harmonics, and faults. These patterns serve as visual signatures that can be analysed to detect and differentiate various power quality events. The key advantage of using Lissajous figures is that they offer a highly sensitive and non-intrusive means of identifying disturbances in real-time, without requiring direct perturbation of the system^1,2^.

Several studies have highlighted the utility of Lissajous figures in power quality monitoring. For instance, Sanz et al. (2015)^3^ demonstrated that Lissajous patterns could accurately distinguish between normal operating conditions and various types of power quality disturbances, including harmonic distortion and voltage sags. The authors noted that Lissajous figures provide a unique method of visualizing power quality issues that is not only intuitive but also effective in real-time applications.

#### Geometric characteristics of Lissajous patterns

The power of Lissajous patterns lies in their geometric characteristics, such as area, skewness, and kurtosis. These parameters, when extracted and analysed, provide valuable insights into the nature of the disturbance and can be used to develop robust indices for detection and localization. Studies have shown that these geometric features exhibit significant variation under different disturbance conditions, making them highly effective for distinguishing between various types of disturbances. For example, the area of a Lissajous figure can be correlated with the magnitude of voltage deviations, while the skewness and kurtosis can provide information about the asymmetry and sharpness of the disturbance, respectively^4^. These geometric features have been successfully used in other power quality monitoring applications, such as harmonic detection and fault classification, where traditional methods often fall short due to the complex nature of disturbances^5^.

#### Adaptive thresholding for detection and localization

A significant challenge in power quality monitoring is the detection of disturbances under dynamic and often unpredictable grid conditions. The proposed approach leverages adaptive threshold values for the indices derived from the geometric characteristics of Lissajous figures. These thresholds are adjusted dynamically based on real-time signal characteristics, ensuring accurate and timely detection of disturbances, even under conditions of overlapping events or grid instability. The use of adaptive thresholds has been explored in previous research on fault detection and localization. Zhang et al. (2016)^6^ proposed a method for fault detection in distribution systems using adaptive thresholding based on real-time signal analysis. Their approach demonstrated enhanced reliability in detecting faults in systems with fluctuating loads and unstable conditions, aligning with the adaptive thresholding approach in the proposed method for power quality disturbance detection.

#### Real-time monitoring and event localization

One of the key strengths of the proposed method is its ability to localize the disturbance source accurately. By analysing the dynamic behaviour of Lissajous figures, the method can identify the exact location of the disturbance within the microgrid. This is crucial for ensuring rapid response and minimizing the impact of power quality issues on sensitive loads and critical infrastructure. Recent advancements in microgrid fault localization have leveraged machine learning and advanced signal processing techniques to enhance detection accuracy and speed. For example, Chen et al. (2017)^7^ demonstrated the application of wavelet transforms combined with machine learning classifiers to identify fault locations in real-time. Similarly, approaches utilizing hybrid systems have been used to improve localization accuracy in large-scale power networks^8^. The proposed method using dynamic Lissajous patterns offers a novel and promising alternative, with the added benefit of being less computationally intensive and more intuitive for real-time applications.

#### Comparative analysis with existing power quality monitoring techniques

The proposed Lissajous-based method outperforms traditional power quality (PQ) monitoring techniques in several key areas, including speed, reliability, and accuracy. Traditional methods such as Fast Fourier Transform (FFT) and Total Harmonic Distortion (THD) analysis, while effective under steady-state conditions, often fail to detect transient disturbances or events with high-frequency components. These limitations are especially pronounced in microgrid environments, where grid conditions are highly dynamic and disturbances frequently overlap^9,10^. Comparative studies have shown that Lissajous pattern-based methods, leveraging geometric features of voltage and current signals, provide more accurate and faster detection. In contrast to FFT and wavelet transform techniques, the Lissajous approach demonstrates superior detection speed and better localization of the disturbance source under varying grid conditions^11^. This makes it highly suitable for real-time PQ monitoring in complex distribution systems. Various techniques for PQ disturbance detection have been proposed, yet they exhibit critical limitations when applied to real-time microgrid scenarios. The Fourier Transform (FT), although widely used for steady-state analysis, performs poorly under transient events such as voltage sags, swells, and short-circuit faults. FT-based techniques also struggle with overlapping disturbances, offering little resolution for harmonic vs. non-harmonic separation in real-time^12^. The Wavelet Transform (WT) offers better handling of non-stationary signals, but its performance is highly dependent on the selection of the mother wavelet and scale parameters. Incorrect choices lead to significant detection errors^13^. In practice, these models often misclassify or miss disturbances in unseen conditions, especially in systems with nonlinearities and renewable energy penetration^14^. These limitations have prompted the use of optimization-based active filtering techniques, such as in^15^, where Artificial Gorilla Troops Optimization improved the performance of FACTS devices in wind-powered power systems. Other works focused on developing new inverter topologies such as split-source^16^ and fifteen-level^17^ inverters, to improve power quality in power systems with renewable energy penetration. Phasor Measurement Units (PMUs), while excellent for synchronized voltage and current measurement, are expensive and limited in detecting fast transient events and localized anomalies and may suffer from cyber-attacks^18^. Their infrastructure overhead makes them unsuitable for widespread deployment in flexible distributed systems. Adaptive thresholding, another conventional approach, often fails to respond accurately in the presence of noise and unpredictable dynamic events, leading to false alarms or missed detections^19^. In contrast, the Lissajous pattern-based approach provides geometric characterization of signal interactions, allowing it to distinguish overlapping or nonlinear events efficiently. It is especially effective in detecting disturbances under conditions of grid instability, high-frequency switching, and fault transients. The dynamic feature extraction enabled by Lissajous trajectories facilitates faster detection and real-time classification, thus overcoming the limitations of threshold-based and purely signal-processing methods. Recent works support the development of integrated signal-processing and ML frameworks. For example^20^, proposed an intelligent PQ detection system combining Multi-Resolution Analysis (MRA) via DWT with an AdaBoost classifier, enhancing robustness to noise and improving classification accuracy. Similarly^21^, introduced a Q-Factor Wavelet Transform (QFWT) method with adaptive tuning, improving detection of non-stationary and complex disturbances. Incorporating optimization algorithms and converter-level control also offers substantial improvements. In^22^, a Jaya-Grey Wolf Optimization method was used to enhance a Three-Level Hybrid Active Filter for EV charging stations, achieving better PQ and compensation of harmonics. Further^23^, demonstrated how fuzzy logic-controlled converters, powered by grid/standalone solar systems, can effectively support PQ in varying conditions.

Moreover, WT suffers from computational complexity, limiting its use in high-speed environments such as smart grids or active filtering applications. These limitations have prompted the use of optimization-based active filtering techniques, such as those proposed in^19,20^, where Neural Network–Honey Badger Optimization and Flower Pollination Optimization have improved the performance of solar-powered UPQC and Shunt Active Power Filters (SAPF). Machine Learning (ML) approaches, including Support Vector Machines (SVM) and Neural Networks (NN), offer promise in classifying and localizing PQ events. However, they require large and diverse datasets to generalize effectively.

### Summary of technical contributions

Power quality disturbances in microgrids can significantly affect the reliability and efficiency of distributed energy systems. The detection and localization of these disturbances in real-time are critical for ensuring the stability and performance of the microgrid. Traditional methods often rely on voltage and current waveforms that can be complex and challenging to interpret. This study presents a novel approach to real-time identification and localization of power quality disturbances by leveraging dynamic Lissajous patterns formed by voltage and current signals. These patterns provide clear, geometrically distinct signatures for various disturbances, including voltage sags, swells, harmonics, and transient faults, making them an ideal tool for disturbance classification.

1. This study presents a novel approach for the real-time identification and localization of power quality disturbances in microgrids, utilizing dynamic Lissajous patterns formed by voltage and current signals. These patterns exhibit distinct geometric shapes in response to various power quality events, including voltage sags, swells, harmonics, and transient faults. By treating these patterns as visual signatures, the method enables clear differentiation between disturbance types.
2. Key contributions include the extraction of geometric characteristics such as area, skewness, and kurtosis from the Lissajous patterns, which are then used to develop robust indices for disturbance detection. The method formulates adaptive threshold values for these indices, allowing for precise event detection and localization without the need for prior knowledge of the microgrid’s network configuration. This is particularly advantageous for real-time monitoring, where network conditions may change rapidly.
3. The proposed technique excels in identifying disturbances even under challenging scenarios, such as overlapping events or grid instability, ensuring accurate localization of the disturbance source. A comparative analysis with existing power quality monitoring techniques demonstrates the superior performance of this method in terms of speed, reliability, and accuracy. Overall, the use of dynamic Lissajous patterns enhances the operational efficiency and resilience of microgrids, offering a powerful tool for advanced real-time power quality monitoring.

The proposed method was selected due to its ability to provide a real-time, interpretable, and computationally efficient solution for detecting and localizing power quality disturbances in microgrids. Unlike traditional waveform-based or frequency-domain techniques, which often require complex signal transformations and prior knowledge of network topology, the use of dynamic Lissajous patterns allows for intuitive visual characterization of voltage-current relationships. By extracting geometric features such as area, skewness, and kurtosis from these patterns, the method achieves high discrimination accuracy across various disturbance types—including voltage sags, swells, harmonics, and transients—without the need for data-intensive training or predefined system models. Additionally, the method’s low computational load and adaptability make it suitable for real-time implementation on embedded or edge devices, aligning well with the dynamic and decentralized nature of modern microgrids.

## Methodology

### Lissajous-based frequency-dependent analysis for power quality event detection

The Lissajous patterns of voltage and current signals exhibit frequency-dependent changes under various power quality (PQ) events. By introducing dynamic modulation and advanced frequency mapping techniques, the method enhances real-time identification and localization of PQ disturbances, including inter-harmonics, frequency drift, and transient frequency deviations. To capture frequency variations in real-time, a time-varying modulation approach is applied to the Lissajous figure. This dynamic modulation adapts the geometric attributes of the Lissajous figure to represent instantaneous changes in frequency. The area and shape of the Lissajous figure evolve dynamically as the frequency of the signal changes, providing insights into transient and steady-state frequency deviations. To capture frequency variations in real-time, a time-varying modulation approach is applied to the Lissajous figure. This dynamic modulation adapts the geometric attributes of the Lissajous figure to represent instantaneous changes in frequency. The area and shape of the Lissajous figure evolve dynamically as the frequency of the signal changes, providing insights into transient and steady-state frequency deviations. For enhanced interpretability, the Lissajous pattern is augmented with frequency-based color-coding, transforming the figure into a frequency map. Each point is color-tagged based on its instantaneous frequency, derived through time-frequency decomposition (e.g., Hilbert transform or STFT).

- **Warm colours (red/orange)**: Represent high-frequency components (e.g., inter-harmonics, switching transients).
- **Cool colours (blue/green)**: Indicate low-frequency variations or normal operating conditions.

This color-coded frequency map enables intuitive visualization of frequency drift, transient spikes, and inter-harmonic propagation—patterns often difficult to detect via conventional spectral methods.

The instantaneous area of the Lissajous figure at time t is calculated using:

1 $$\:{A}_{v-i}=\underset{i(\tau\:=t-T)}{\overset{i(\tau\:=t)}{\int\:}}v\left(\tau\:\right)di\left(\tau\:\right)$$

where:

- v(τ) Voltage signal.
- i(τ)Current signal.
- T is Time interval of integration.
- t is Current time instance.

To detect changes, the area at the current time t is compared with the area at the previous time t − Δt where Δt is the time step:

2 $$\:{A}_{v-i}(t-\varDelta\:t)=\underset{i\left(\tau\:=t-T-\varDelta\:t\right)}{\overset{i\left(\tau\:=t-\varDelta\:t\right)}{\int\:}}v\left(\tau\:\right)di\left(\tau\:\right)$$

### Frequency mapping of lissajous patterns for visualization

To visualize the frequency variations dynamically, the Lissajous figure is enhanced with frequency-based color-coding. The instantaneous frequency is determined using advanced signal processing techniques, and each point of the Lissajous figure is assigned a colour corresponding to its frequency at that moment. This creates a dynamic frequency map where:

- High frequencies are represented with warmer colours (e.g., red, orange).
- Low frequencies are depicted with cooler colours (e.g., blue, green).

The color-coded Lissajous figure provides an intuitive representation of frequency drift, inter-harmonics and transient events.

Frequency-Dependent Similarity Index for Lissajous Figures.

The similarity index (SI) is modified to incorporate frequency-dependent changes:

2 $$\:SI\left(t\right)=1-\frac{\left|{A}_{v-i}\left(t,f\right)-{A}_{v-i}\left(t-\varDelta\:t,f\right)\right|}{max\left\{{A}_{v-i}\left(t,f\right)-{A}_{v-i}\left(t-\varDelta\:t,f\right)\right\}}$$

where Δt is the time difference between successive intervals.

Under normal operating conditions, the successive areas of the Lissajous figure are nearly identical, resulting in an SI value close to 1. During a PQ disturbance, the area changes significantly, causing the SI to deviate from 1. By choosing an appropriate threshold for the SI, the disturbance can be reliably detected. While the conventional SI provides reliable detection of PQ disturbances in static conditions, its performance diminishes when frequency variations, such as inter-harmonics, frequency drift, or transient frequency deviations, are introduced. The conventional SI is derived purely from the geometric area and does not account for the influence of frequency changes on the Lissajous pattern. During events involving overlapping PQ disturbances and frequency drift, the similarity index may yield values close to the threshold, leading to false positives or missed detections. Modern microgrids experience rapid changes in frequency due to distributed energy sources and varying load profiles, which are not adequately captured by the area-based SI. To address the aforementioned challenges, a frequency-dependent Similarity Index (FDSI) is proposed. This new formulation incorporates instantaneous frequency variations into the calculation of the Lissajous figure’s area, enabling the detection of events influenced by frequency-related disturbances. By dynamically modulating the Lissajous figure and integrating frequency-dependent attributes, the proposed FDSI overcomes the limitations of the conventional method, providing improved sensitivity and robustness in detecting PQ disturbances under dynamic grid conditions. This frequency-weighted SI enhances detection sensitivity for frequency-related disturbances. By combining dynamic modulation and frequency mapping of Lissajous figures, the method provides precise detection and localization of PQ disturbances. This approach is particularly effective for identifying events involving frequency drift or inter-harmonics ensuring robust monitoring of power quality in modern microgrids. This innovative Lissajous-based frequency-dependent analysis empowers utilities to track, visualize, and mitigate frequency-related PQ issues, enhancing grid stability and reliability.

### Dynamic modulation of SI

Incorporate time-varying modulation to enhance sensitivity during rapidly changing PQ events. Modulate SI(t) dynamically to capture real-time variations:

3 $$SI_{dynamic}(t) = FWSI(t).\left(1 + \propto \frac{{df(t)}}{{dt}}\right)$$

Where FWSI is a frequency-dependent weighting function W_f_(t) to account for frequency variations, such as inter-harmonics, transient deviations, and frequency drift.

4 $$FWSI(t)=1-\:\frac{\left|{W}_{f}\left(t\right){A}_{v-i}\left(t\right)-{W}_{f}\left(t-\varDelta\:t\right)-{A}_{v-i}(t-\varDelta\:t)\right|}{max\left\{{W}_{f}\left(t\right){A}_{v-i}\left(t\right)-{W}_{f}\left(t-\varDelta\:t\right)-{A}_{v-i}(t-\varDelta\:t)\right\}}$$

where:

W_f_(t): Weighting function based on instantaneous frequency.

A_v−i_(t) is Area of the Lissajous figure at time t.

$\:\frac{df\left(t\right)}{dt}$ Rate of change of frequency.

α: Modulation factor.

**Summary of parameters**

**Table:** 

Parameter	Purpose	Typical value range
W_f_(t)	Frequency weighting function	Gaussian or sigmoid function
T	Time interval for Lissajous area calculation	10–50 ms (system dependent)
Δt	Sampling interval	0.1–1 ms (system dependent)
α	Modulation factor	0.1–1 (experimentally determined)

By carefully selecting and tuning these parameters, the Dynamic SI can achieve high accuracy, robustness, and adaptability for PQ disturbance detection and localization in real-time systems. Selection of α in SI_dynamic_(t) SI(t). The parameter α serves as a modulation factor that determines the sensitivity of the dynamic similarity index SI_dynamic_(t) to frequency changes. Selecting an appropriate value for α\alpha is crucial for balancing the system’s responsiveness to power quality (PQ) disturbances and its robustness against noise or normal operational variations. A small value of α minimizes the modulation effect, ensuring that SI_dynamic_(t) remains stable during minor fluctuations, such as those caused by steady-state conditions or measurement noise. However, this might reduce sensitivity to transient PQ events, particularly those involving rapid frequency drifts or interharmonics. Conversely, a larger value of α increases the sensitivity of the index, enabling the detection of subtle disturbances. Yet, excessive sensitivity may lead to false positives, where normal conditions are misclassified as PQ disturbances. To select α, a systematic approach is recommended. Begin with a moderate baseline value (e.g., 0.1 or 0.5) and evaluate the system’s performance using a representative dataset containing diverse PQ scenarios. Gradually adjust α\alpha while monitoring its impact on key performance metrics, such as detection accuracy, false positive rate, and response time. The optimal value is typically one that maximizes detection accuracy while maintaining an acceptable false positive rate. In applications requiring high precision, optimization techniques can further refine α\alpha. For instance, grid search can systematically explore discrete α\alpha values, while advanced methods like genetic algorithms or particle swarm optimization can dynamically adapt α\alpha based on real-time feedback. The chosen value should reflect the operational characteristics of the grid, ensuring that the system adapts effectively to both rapid disturbances and steady-state conditions.

**Fig. 1:**
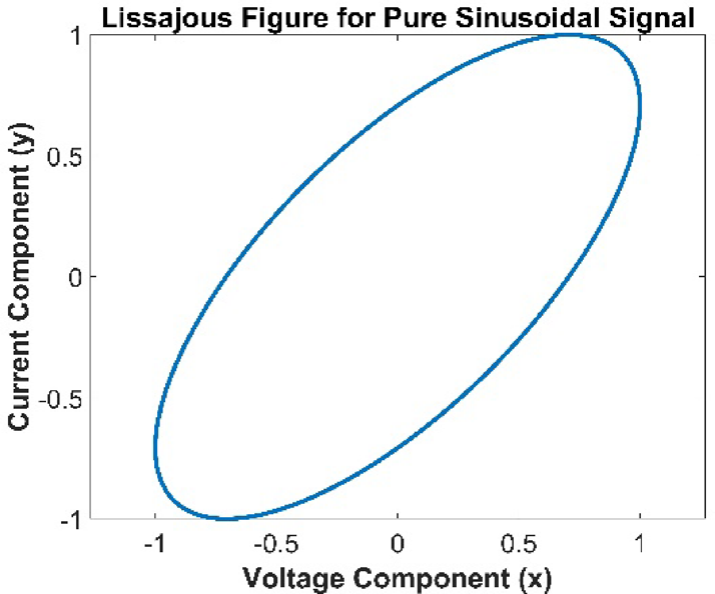
Lissajous Figure of pure sinusoidal signal.

The elliptical shape of the Lissajous figure for sinusoidal signals, as observed through the Similarity Index (SI) framework, is a direct consequence of the periodic nature of the signals and their harmonic relationship. The area of the Lissajous figure, A_v−i_(t), is calculated using the integral of voltage v(t) with respect to current i(t). For sinusoidal waveforms with the same frequency but potentially different amplitudes and phases, the relationship between v(t) and i(t) forms a closed-loop curve. The sinusoidal signals ensure smooth and periodic variations in the area, resulting in successive areas, A_v−i_(t) and A_v−i_(t − Δt), that are nearly equal under normal conditions. This behaviour causes the SI, which measures the similarity between these areas, to remain close to 1. The elliptical shape specifically arises due to the harmonic interplay between the voltage and current signals. The ratio of their amplitudes determines the elongation of the ellipse along its axes, while the phase difference between the signals tilts the ellipse, creating its characteristic symmetry. This harmonic consistency ensures that the figure remains elliptical for pure sinusoidal signals as shown in Fig. 1. However, challenges arise when the signals deviate from this ideal condition. Interharmonics, transient events, or frequency drift can distort the symmetry of the Lissajous figure and introduce abrupt changes in the area, reducing the reliability of the SI for accurate detection. To overcome these limitations, enhancements such as dynamic modulation of the SI or frequency-dependent weighting functions can be incorporated. These modifications ensure that the SI adapts to the complexities of non-sinusoidal signals, maintaining accuracy in detecting power quality disturbances during events such as interharmonics or transient frequency deviations.

### Power quality events and location identification

As per the proposed technique i.e. Lissajous figures provides a visually rich and quantitative way to identify power quality (PQ) events based on the Similarity Index (SI) and associated metrics. Each event introduces distinct changes in the shape, amplitude modulation, and color-coded z-dimension, which is directly linked to a custom event metric. By observing these variations, PQ events such as sag, swell, harmonics, flicker, and transients can be effectively identified. The SI value, derived from every event, quantifies the similarity between successive areas of the Lissajous figure. Events with minimal PQ disturbances maintain high SI values (close to 1), indicating consistent harmonic behaviour. Conversely, significant disturbances such as transients, flicker, or notches result in reduced SI values, highlighting deviations from the sinusoidal baseline. By integrating the z-metric and SI, the program offers a comprehensive framework for classifying and detecting PQ events with high accuracy and clarity The centroid of each Lissajous figure is computed as the mean value of the voltage (x-axis) and current (y-axis) components. This represents the average position of the curve in the voltage-current plane and highlights the central tendency of the signal. By comparing the centroid position for different events, shifts in the PQ signal behaviour due to disturbances can be identified. The bounding area is calculated as the product of the ranges of the voltage and current components, effectively forming a rectangular approximation around the Lissajous figure. This metric reflects the spatial extent of the figure, where larger areas often correspond to events like swell or overvoltage, and smaller areas indicate events such as sag or interruptions. Eccentricity, derived from the covariance matrix of the voltage and current components, measures the elliptical shape of the Lissajous figure. An eccentricity value close to 1 indicates a more elongated shape, which may occur during harmonic distortions or flicker events. Values closer to 0 suggest a more circular figure, often seen in balanced signals. For each PQ event, the centroid provides the location shift, the bounding area reflects the signal’s magnitude and deviation, and eccentricity indicates signal asymmetry or distortion. Comparing these metrics against those of the pure sinusoidal signal (baseline) reveals deviations in centroid location, bounding area, and eccentricity, allowing for the identification and characterization of PQ events. These metrics are further annotated on the Lissajous plots for visual validation. Each PQ event produces unique metrics that serve as a fingerprint to identify and locate disturbances in the signal space. By examining centroid shifts from the baseline, bounding area changes, and eccentricity variations, the type and severity of PQ disturbances can be pinpointed. This integrated approach of visual representation and metric-based analysis offers a robust method for detecting and locating PQ events in electrical systems.

### Implementation of the proposed method

**Fig. 2:**
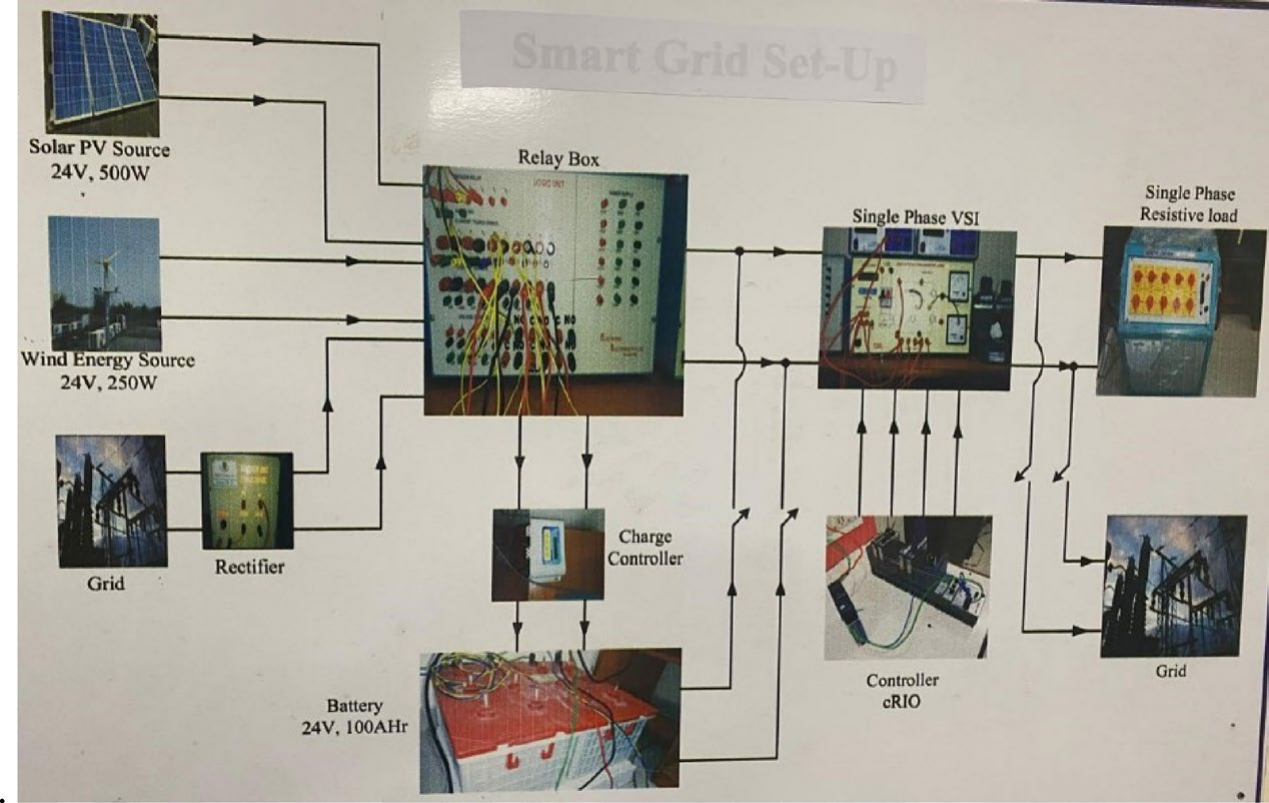
Test system

The smart grid setup shown in the Fig. 2 integrates multiple renewable energy sources, including a 24 V, 500 W solar PV source and a 24 V, 250 W wind energy source, alongside a 24 V, 100Ah battery storage system and a grid connection. These energy sources are connected to a relay box that manages their inputs and coordinates the flow of energy. The energy from renewable sources and the grid is rectified, regulated by a charge controller, and stored in the battery. The system also includes a single-phase voltage source inverter (VSI), which converts DC power from the battery to AC power suitable for supplying a single-phase resistive load. A controller (cRIO) monitors and manages the system’s operation, ensuring efficient energy utilization and balancing between sources, storage, and loads. This setup demonstrates the implementation of a smart grid by effectively integrating renewable energy, storage, and traditional grid sources to supply reliable power while maintaining flexibility and efficiency.

**Fig. 3:**
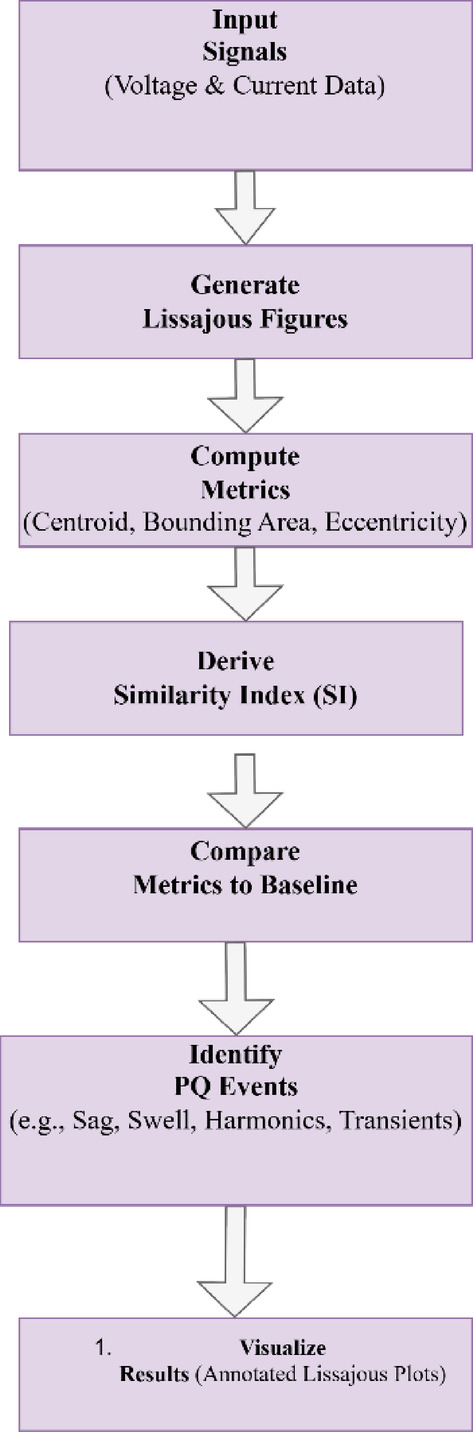
Flowchart.

As shown in Fig. 3., the proposed flowchart for detecting and locating Power Quality (PQ) events using Lissajous figures begins with acquiring input signals, including voltage and current data from the power system. These signals are processed to generate Lissajous figures, which visually represent the relationship between voltage and current. Metrics such as centroid, bounding area, and eccentricity are computed from the Lissajous figures to quantify their spatial and geometric properties. The Similarity Index (SI) is then derived to measure deviations from the baseline sinusoidal behavior. These computed metrics and SI values are compared to the baseline to identify deviations indicative of PQ events. Based on these deviations, events such as sag, swell, harmonics, flicker, and transients are classified. Finally, annotated Lissajous plots, enriched with calculated metrics, are visualized to provide a clear and comprehensive understanding of the PQ disturbances. This integrated process ensures accurate detection, classification, and visualization of PQ events in electrical systems.

## Result analysis

The Smart Grid experimental setup integrates renewable and conventional energy sources with energy storage and load management systems. It comprises a Solar PV Source with a specification of 24 V, 500 W, and a Wind Energy Source rated at 24 V, 250 W. A Grid Supply provides additional power through a Rectifier that converts AC to DC for system compatibility. The energy storage unit features a Battery rated at 24 V, 100 Ah, regulated by a Charge Controller. The control and switching operations are managed by a Relay Box, ensuring smooth power flow to the Single-Phase Voltage Source Inverter (VSI), which delivers AC power to a Single-Phase Resistive Load. The load and grid are connected via a Controller cRIO for monitoring and control. The transmission line components include resistance (R), inductance (L), and capacitance (C) values to emulate realistic conditions in a distribution network. The values for R, L, and C are as follows: R = X Ω, L = Y mH, and C = Z µF (fill in actual values if known). This setup facilitates the study of load balancing, renewable energy integration, and energy storage performance, making it ideal for smart grid applications. The proposed has been implemented under 17 types of Power Quality (PQ) events.

**Table 1:** Values of different parameters of lissajous based proposed methods.

Metrics	Pure Signal	Sag	Swell	Interruption	Harmonic	Flicker	Notch	Transient	DC off sets	Frequency driff	Interharmonics	Oscillations	Voltage Imbalance	Current Imbalance	Harmonic Flicker	Overvoltage	Under Voltage
Centroid	[0.0007064, 0.000999]	[0.0007064, 0.0010989]	[0.0007064, 0.0011988]	[0.0007064, 0.0012987]	[0.0007064, 0.0013986]	[0.0007064, 0.0014985]	[0.0007064, 0.0015984]	[0.0007064, 0.0016983]	[0.0007064, 0.0017982]	[0.0007064, 0.0018981]	[0.0007064, 0.001998]	[0.0007064, 0.0020979]	[0.0007064, 0.0021978]	[0.0007064, 0.0022977]	[0.0007064, 0.0023976]	[0.0007064, 0.0024975]	[0.0007064, 0.0025974]
Bonding Area	4	4.769	5.653	6.0363	7.6875	8.8313	10.0556	11.358	12.74	14.203	15.7447	17.3715	19.0783	20.8651	22.7318	24.6784	26.7051
Eccentricity	0.91031	0.907	0.8994	0.8875	0.87049	0.85028	0.827	0.8026	0.7765	0.74997	0.72325	0.6968	0.67093	0.64584	0.62168	0.59854	0.57646

The observations from Table 1 reveal significant variations in signal characteristics across different power quality events. The centroid values progressively increase from the pure signal ([0.0007064, 0.000999]) to disturbances such as DC offsets ([0.0007064, 0.0017982]) and voltage imbalance ([0.0007064, 0.0021978]), indicating a shift in signal properties as the disturbance severity escalates. Similarly, the bonding area, which represents the energy spread or complexity in the signal, grows steadily from 4 for a pure signal to 12.74 for DC offsets and further to 26.7051 for under voltage conditions. This trend highlights the increasing energy complexity introduced by disturbances. Eccentricity values, on the other hand, decrease with disturbance severity, starting at 0.91031 for a pure signal and dropping to 0.7765 for DC offsets and 0.57646 for under voltage, signifying a shift from a symmetrical distribution to a more elongated one. Events like harmonic flicker and notching show intermediate centroid shifts and bonding area increases, reflecting moderate deviations compared to sag, swell, and harmonic disturbances. Overall, as disturbances become more severe or complex, centroids increase, bonding areas expand, and eccentricities decrease, showcasing the progressive deviation of signal geometry and energy distribution from the baseline pure signal. These metrics effectively characterize and differentiate power quality events, aiding in fault detection and system evaluation.

**Fig. 4:**
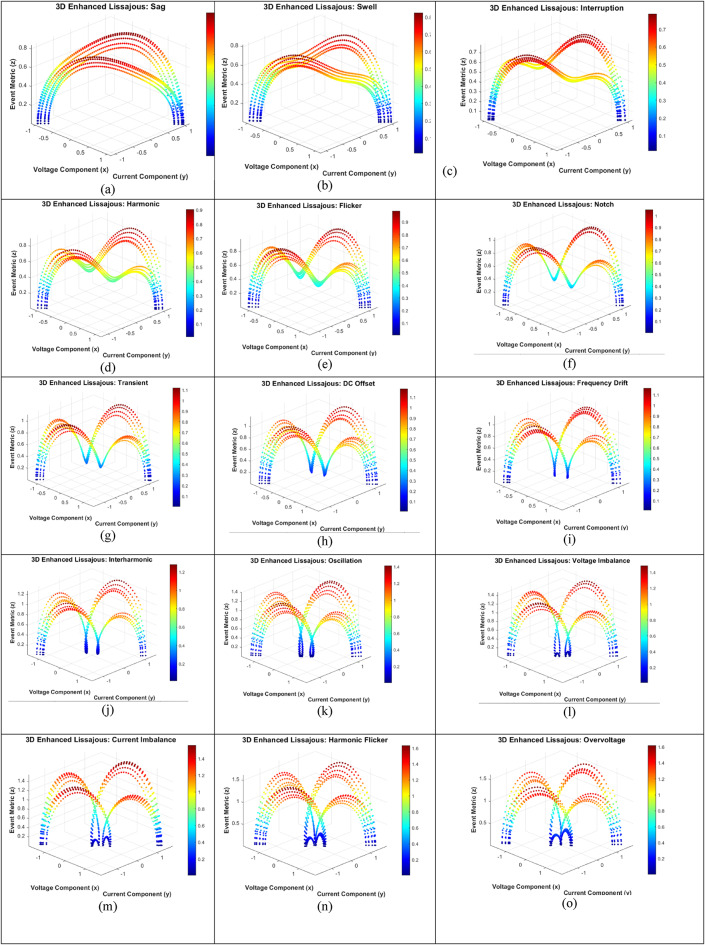

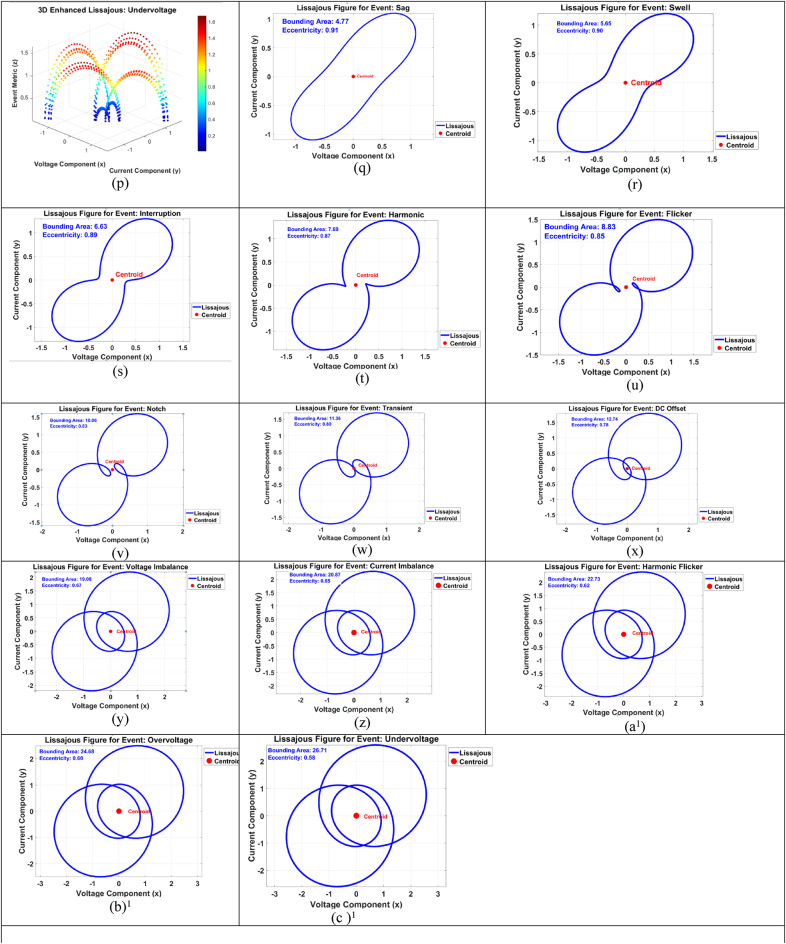
Different Lissajous figures under different conditions.

The generated 3D Lissajous figures (shown in Fig. 4(a) to 4 (p)) dynamically visualize power quality (PQ) events by illustrating time-varying relationships between voltage and current signals, enhanced using the SI dynamic index to quantify event-specific signal behaviour. The pure sinusoidal Lissajous figure represents a stable system with no distortions, serving as a baseline reference. Sag and swell events alter the amplitude dynamics of the Lissajous patterns, resulting in visibly smaller or larger trajectories, indicating voltage level changes captured by the SI index. Interruptions drastically reduce the figure’s scope, reflecting signal loss, while harmonic distortion introduces ripples to the Lissajous trajectory due to added high-frequency components, with the SI index highlighting these variations. Flicker dynamically modulates the Lissajous figure’s trajectory, simulating repetitive oscillations, while notch and transient events are visualized as sharp, high-amplitude deviations along the zz-axis of the 3D Lissajous figure, representing short-duration disturbances quantified by the SI index. DC offset shifts the centroid of the Lissajous pattern vertically, showcasing signal imbalance detected by the index. Frequency drift distorts the figure’s smooth trajectory, highlighting gradual instability, while interharmonics create complex, overlapping Lissajous patterns due to interacting frequencies. Voltage and current imbalances asymmetrically stretch or compress the Lissajous figure, representing unequal signal distributions effectively described by the SI dynamic index. Harmonic flicker combines ripples and oscillations in the Lissajous figure, capturing interactions between harmonics and flicker. Overvoltage and undervoltage proportionally expand or contract the Lissajous patterns, indicating changes in dynamic signal magnitude.

Lissajous figures, as illustrated in Fig. 4(q) to 4(c)^1^, serve as a sophisticated visual and analytical tool for examining the dynamic relationship between two periodic signals—typically voltage and current—in power systems. These figures are generated parametrically by plotting voltage on the X-axis and current on the Y-axis. Under ideal operating conditions—where voltage and current have identical frequencies, equal amplitudes, and a stable phase difference—the resulting shapes include well-defined geometries such as circles, ellipses, or straight lines. Deviations from these standard forms reveal critical insights into the presence of power quality (PQ) disturbances.

A perfect ellipse, for instance, indicates a stable sinusoidal relationship, whereas irregular or distorted shapes highlight anomalies such as harmonics, transients, or imbalances. Several quantifiable metrics enhance the diagnostic utility of Lissajous figures. The **centroid**, representing the geometric centre of the pattern, is calculated as the mean of the voltage and current data points. In an undisturbed system, the centroid aligns with the origin (0,0); however, signal imbalances or DC offsets displace the centroid, offering immediate visual cues. For example, a DC offset in the current shifts the centroid along the Y-axis, signalling the presence of a disturbance.

Another valuable metric is the bounding area of the figure, which encapsulates the amplitude dynamics of the signals. Power quality events such as voltage sags or swells manifest as contractions or expansions in the bounding area. An increase may suggest harmonic distortions due to high-frequency components amplifying waveform oscillations. **Eccentricity** serves as a measure of signal asymmetry. While a near-circular figure implies balanced amplitudes and a 90° phase difference, elongation due to phase or amplitude imbalances (e.g., sags or swells) results in higher eccentricity, making it a sensitive detector of such events.

Moreover, complex PQ issues such as harmonics and transients introduce additional geometric complexities. Harmonics may add ripples or lobes to the Lissajous curve, while transients cause abrupt deviations, transforming smooth loops into jagged paths. Interharmonics and frequency drifts further deform the figure, leading to open-loop or asymmetric shapes indicative of synchronization failures or system instabilities. These geometric transformations offer a rich feature space for signal classification.

**Fig. 5:**
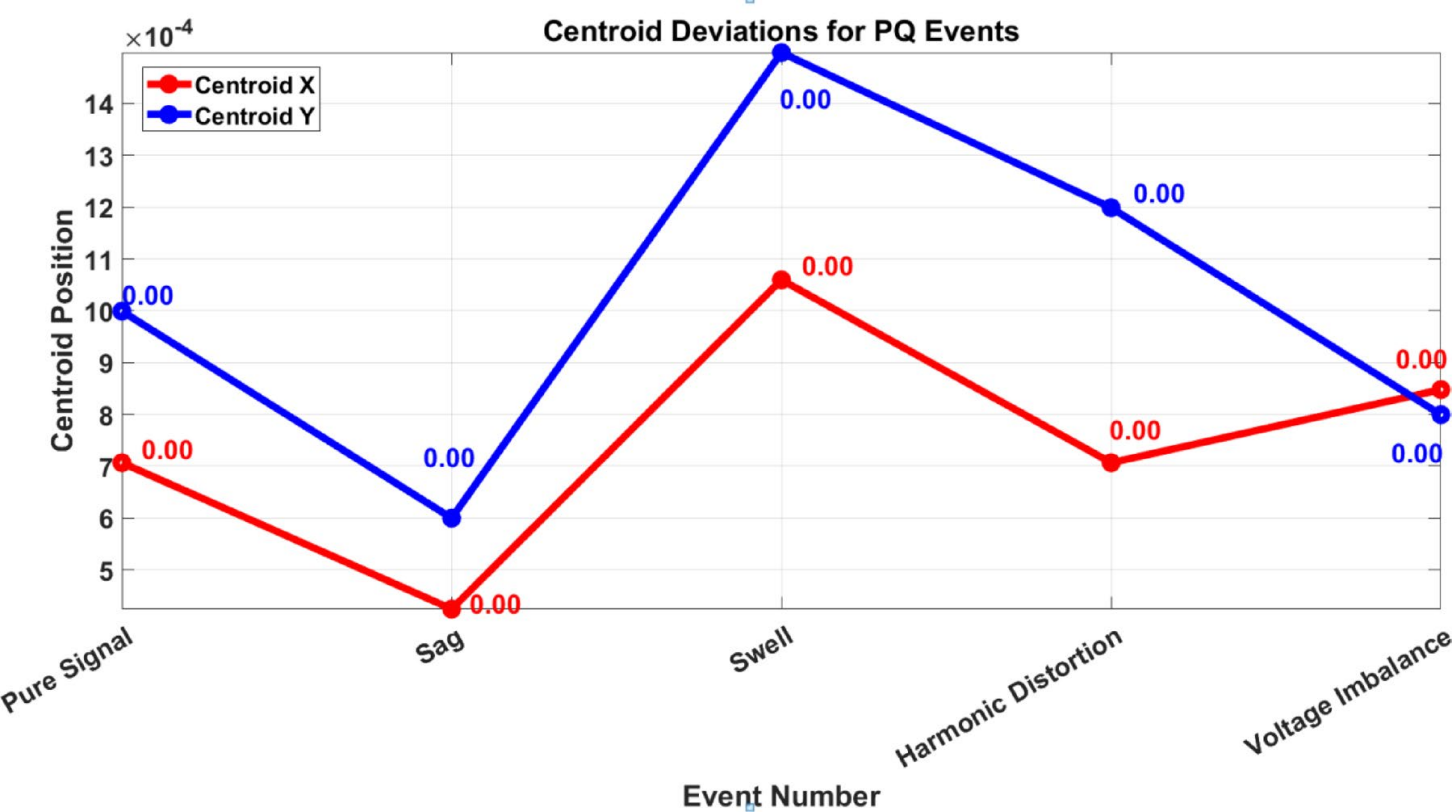
Graph of Centroid Point vs. Event number.

The potential of these metrics is further validated by Fig. 5, which demonstrates centroid deviations across different PQ events. In this visualization, the X-axis corresponds to specific events (e.g., pure signal, sag, swell, harmonic distortion, voltage imbalance), while the Y-axis tracks centroid positions in both X (red) and Y (blue) directions. For the pure signal, the centroid remains stable at (0,0), serving as a reference. As disturbances occur, such as in the case of a sag or swell, the centroid shifts accordingly. For instance, a voltage swell, which significantly alters the signal amplitude, causes a larger deviation than a minor imbalance or sag.

This data is supported numerically by Table 2, which lists the corresponding Euclidean distances between the disturbed signal centroid and the reference point. For example, swell results in the highest distance (0.7071), followed by harmonic distortion (0.5657), voltage imbalance (0.3536), and sag (0.2828). These values correlate strongly with the centroid deviations shown in Fig. 5, affirming the robustness of the feature extraction method.

The consistency of centroid positioning—particularly the return to (0,0) under undisturbed conditions—demonstrates the system’s normalization capability and resilience. Stable centroids across events enhance classification reliability by minimizing noise-induced deviations. When integrated with machine learning classifiers, metrics such as centroid, bounding area, and eccentricity provide a powerful framework for real-time detection, localization, and classification of PQ events. This approach not only improves monitoring capabilities but also supports predictive maintenance strategies, contributing to the operational efficiency and resilience of modern power system.

### Centroid deviations for power quality events and their correlation with Euclidean distance metrics

The provided figure, titled *“Centroid Deviations for Power Quality (PQ) Events*,*”* visually represents centroid shifts corresponding to various PQ disturbances, aligning with the numerical analysis presented in Table 2; Fig. 5. The x-axis denotes different PQ events, including Pure Signal, Sag, Swell, Harmonic Distortion, and Voltage Imbalance, while the y-axis represents the centroid coordinates in both X (red) and Y (blue) directions, illustrating the variations in feature space due to different disturbances. The correlation between centroid deviations and the Euclidean distance metrics in Table 2 is evident, as larger centroid shifts correspond to higher distance values, reflecting the severity of PQ disturbances. For instance, the substantial centroid deviation observed in the Y-direction for Swell corresponds to the highest Euclidean distance (0.7071), while Harmonic Distortion and Voltage Imbalance exhibit noticeable centroid shifts, aligning with their respective distances (0.5657 and 0.3536). The Pure Signal remains stable at (0,0), reaffirming its zero-distance reference and indicating the absence of disturbances. This consistency highlights the effectiveness of the feature extraction method in normalizing variations, ensuring an optimized and resilient classification system. The alignment of centroid deviations with Euclidean distance values underscores the robustness of the proposed approach in PQ event detection and classification, validating its reliability for real-time monitoring and power system analysis.

**Table 2:** Euclidean distances for PQ events:.

PQ event	Distance
Pure Signal	0.0000
Sag	0.2828
Swell	0.7071
Harmonic Dist.	0.5657
Voltage Imbalance	0.3536

**Fig. 6:**
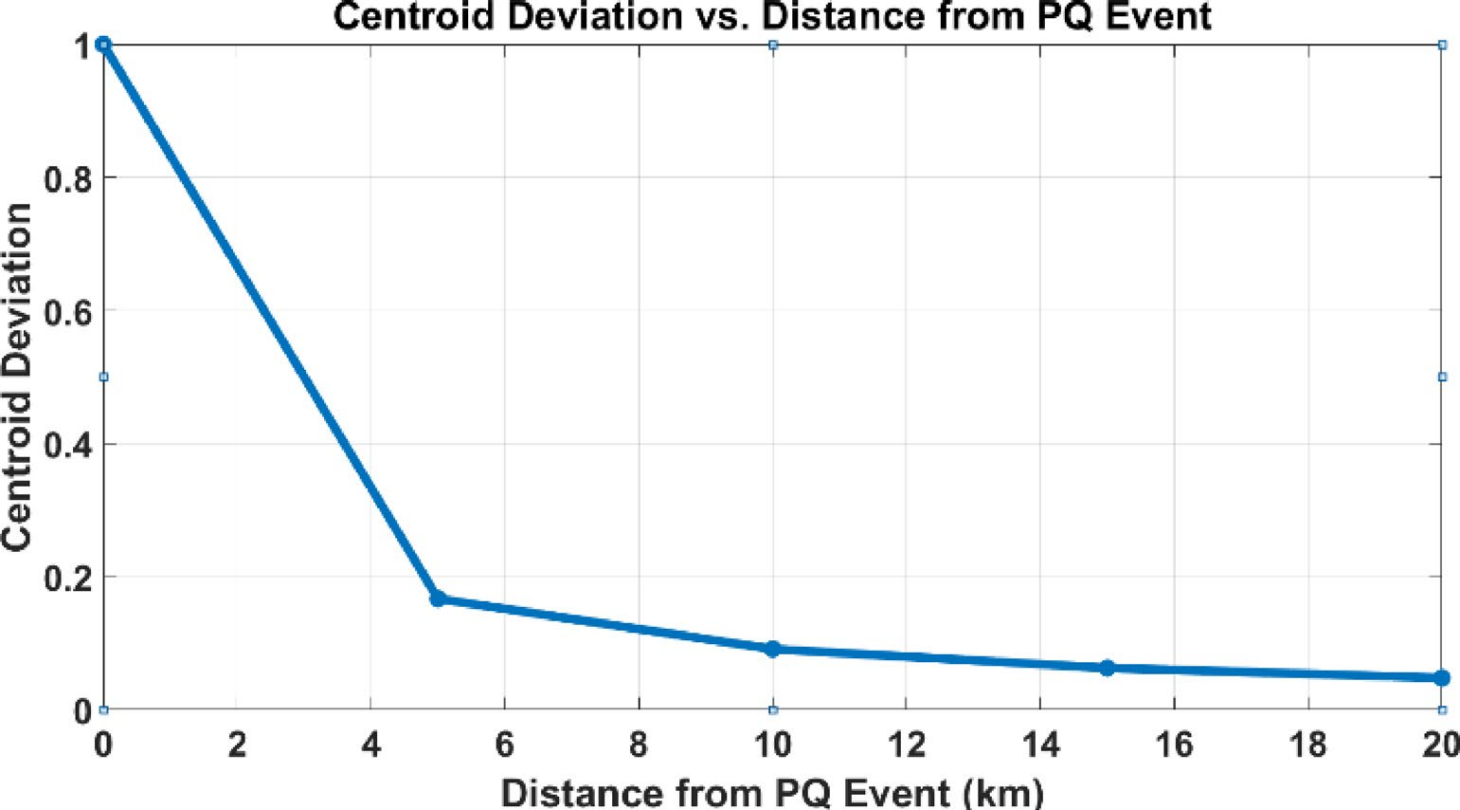
Distance vs. Centroid deviation.

Table 2 provides the distance metrics for various power quality (PQ) events in relation to a pure signal. These metrics quantify the deviation of each disturbance type from the ideal, undisturbed signal. The “Pure Signal” serves as the baseline with a distance value of 0, indicating no disturbance. For other events, the distance values vary, reflecting the extent of deviation caused by each disturbance. The voltage sag, with a distance of 0.2828, shows a moderate deviation, indicating a slight decrease in voltage. The voltage swell, with a distance of 0.7071, represents a more significant deviation, indicating a substantial increase in voltage. Harmonic disturbances, which introduce higher frequency components, have a distance of 0.5657, showing a noticeable but less extreme deviation. Voltage imbalance, with a distance of 0.3536, reflects a moderate deviation, indicating an uneven distribution of voltage across phases. These distance values serve as essential indicators for distinguishing between different PQ events, aiding in their detection and classification for real-time monitoring and analysis.

The curve shown in Fig. 6 represents the relationship between centroid deviation and distance from a Power Quality (PQ) event in a power system. As shown, the centroid deviation is highest at the monitoring point closest to the PQ event and progressively decreases with increasing distance from the event. This inverse relationship occurs because the impact of the PQ disturbance, such as a voltage sag or harmonic distortion, diminishes as it propagates through the power system due to impedance, damping, and network topology. The sharp decline in deviation near the event indicates its localized nature, while the gradual tapering off reflects the distributed effects on the system. By analysing this curve, the location of the PQ event can be identified—typically corresponding to the monitoring point with the maximum centroid deviation. This analysis provides a systematic approach to event localization, aiding in fault diagnosis and system reliability improvement.

## Experimental verification

**Fig. 7:**
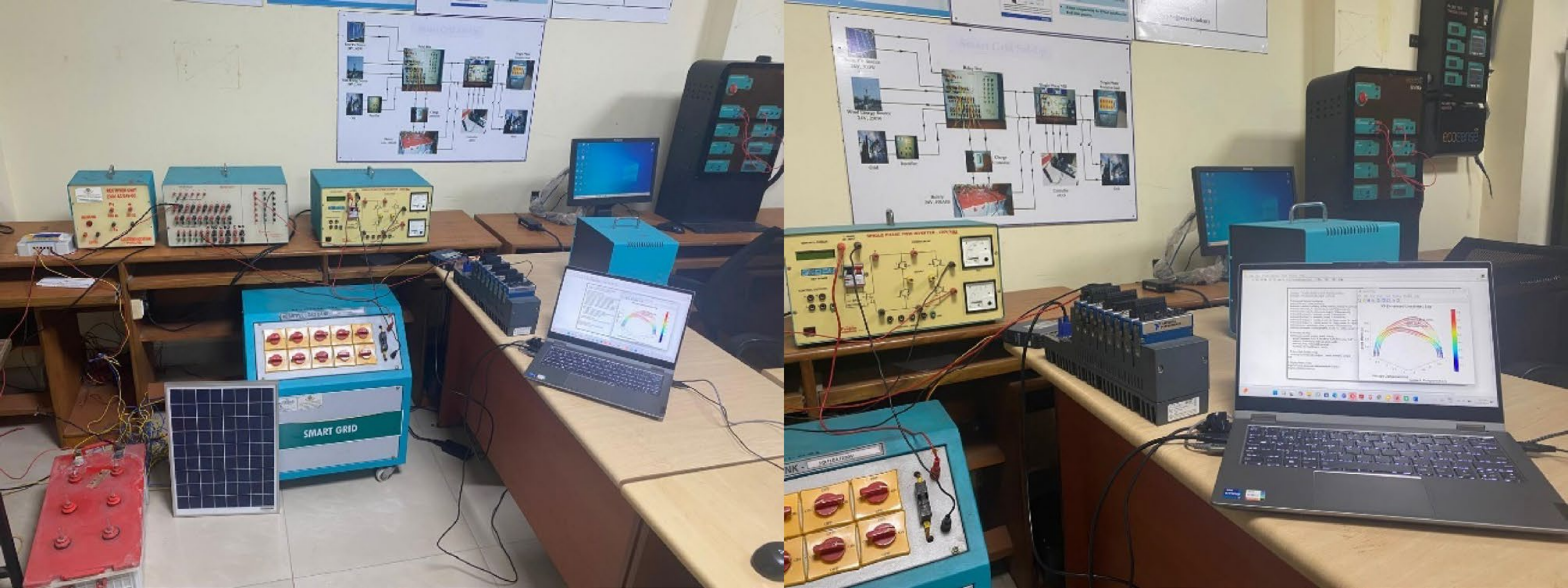
Experimental Setup.

The experimental setup shown in Fig. 7 demonstrates a smart grid system integrating solar PV (24 V, 500 W), wind energy (24 V, 250 W), and grid power. The relay box ensures safe switching and protection of energy flow between the components, while the rectifier converts AC outputs from the wind and grid sources into DC, which, along with the solar PV output, is fed into the charge controller. The charge controller regulates the charging and discharging of a 24 V, 100Ah battery, maintaining optimal energy storage for system operation. The stored energy powers a single-phase voltage source inverter (VSI), converting DC into AC to supply a resistive load or feed back into the grid. The cRIO controller supervises and manages real-time energy flow, ensuring load balancing and system stability.

The laboratory setup includes advanced equipment integrated with LabVIEW for real-time data acquisition and analysis, with specific model numbers detailed below. The National Instruments USB-6356 DAQ serves as the central interface, featuring a high-speed, multifunction data acquisition system with 16-bit resolution and a sampling rate of up to 1.25 MS/s. It collects signals from the connected equipment and transmits them to LabVIEW for analysis. The Technovate Power System Trainer Model PST-2000 simulates various power system scenarios such as faults, harmonic distortions, and load variations, generating real-time data for LabVIEW-based visualization and analysis. Voltage and current measurements are captured using LEM LA-55-P Current Sensors and LV 25-P Voltage Sensors, which interface directly with the DAQ for precision monitoring. A Texas Instruments TMS320F28379D DSP Kit performs real-time signal conditioning, including filtering and amplification, before transmitting pre-processed signals to LabVIEW for proposed Lissajous method. The setup also includes ABB REF615 Protection Relays, which are connected to LabVIEW via the DAQ for monitoring protection events and triggering alarms in fault scenarios. This integration enables a comprehensive platform for power quality analysis and fault diagnosis. Experimental verification involves assessing the performance of energy generation and integration under varying conditions, monitoring battery efficiency, evaluating the inverter’s output quality, analysing grid interaction, and validating the controller’s ability to manage dynamic scenarios like load changes or source failures. The setup effectively demonstrates smart grid capabilities in integrating renewable sources, optimizing energy storage, and ensuring a reliable energy supply.

Table 3 provides a visual summary of the comparative analysis between proposed method and other methods available in the literature. This includes different parameters such as detection approach, event localization, adaptability to instability, real time performance, and robustness to noise. As stated in the table, the proposed approach is superior to other methods in almost all aspects, It utilizes a more robust detection method, is faster and less resource intensive, is able to localize events, can operate under grid instability or present of noise and provides excellent real-time performance. In light of above simulation and experimental validations it is established that the proposes approach is superior and shows much better performance.

**Table 3:** Comparative analysis.

Aspect	Proposed dynamic Lissajous patterns method	Fourier Transform (FT)	Wavelet Transform (WT)	Support Vector Machine (SVM) based detection	Principal Component Analysis (PCA) with ML
Event Detection Approach	Identifies and localizes disturbances using dynamic Lissajous patterns formed by voltage and current.	Uses frequency domain analysis to detect harmonics, sags, and swells by transforming the signal.	Utilizes time-frequency analysis for transient and non-stationary disturbance detection.	Detects power quality disturbances based on feature extraction and classification via SVM.	Reduces dimensionality and detects disturbances by analyzing principal components in power signals.
Geometric Features for Analysis	Geometric characteristics such as area, skewness, and kurtosis of Lissajous patterns.	Frequency peaks and harmonic content analysis.	Time-frequency decomposition, wavelet coefficients.	Statistical features such as mean, variance, and energy extracted from the signal.	Feature vectors based on variance and covariance of the principal components.
Event Localization	Provides precise event localization by analyzing the dynamic shape of Lissajous patterns.	No direct localization, relies on overall disturbance characteristics across the system.	Localization is possible but challenging; often used in combination with other techniques.	Localization depends on the grid topology and classification results.	PCA does not inherently localize disturbances but may improve detection across multiple sensors.
Handling Overlapping Disturbances	Effectively identifies and differentiates overlapping disturbances (e.g., voltage sag and harmonics).	Fourier transform is less effective for overlapping events, leading to difficulties in clear separation.	Can decompose complex signals, but overlaps may be difficult to resolve without advanced techniques.	SVM-based methods can classify overlapping disturbances if properly trained, but may struggle in real-time.	PCA works well for detecting disturbances but may struggle with overlapping events unless combined with ML classifiers.
Adaptability to Grid Instability	Works well in unstable grid conditions without needing prior knowledge of grid configuration.	Less adaptive to grid instability; relies on steady-state conditions.	More adaptable to transients and dynamic disturbances.	SVM may need retraining or more features to handle grid instability.	PCA requires careful tuning to adapt to unstable grid conditions, often needing additional features.
Threshold Adaptation	Adaptive thresholds for event detection based on the Lissajous pattern characteristics.	Uses fixed or manually set thresholds for event detection.	Uses multi-scale decomposition, but thresholds for detection need manual calibration.	SVM thresholds are typically static unless retrained with updated data.	Thresholds for PCA may be adapted through post-processing, but it requires additional machine learning steps.
Real-Time Performance	Highly real-time performance, capable of detecting and localizing events with high speed.	Generally slower, as FT requires signal transformation and analysis of frequency spectra.	Time-frequency decomposition can be computationally expensive, slowing real-time detection.	Real-time performance depends on SVM model complexity and size of the training dataset.	PCA may not be real-time without fast computation techniques or prior feature extraction.
Accuracy in Event Classification	High accuracy in detecting and classifying disturbances with minimal false positives due to unique pattern recognition.	Lower accuracy in detecting complex events like transients, overlapping disturbances, and nonlinear phenomena.	High accuracy in transient events but may miss non-stationary faults or disturbances with low energy.	High classification accuracy if well-trained on diverse disturbance data, but might struggle with edge cases.	Can provide good accuracy in classifying disturbances when combined with ML, but PCA alone might miss complex events.
Application to Complex Events	Efficient in detecting complex power quality disturbances, such as transient faults and simultaneous events.	Struggles with complex, transient, or simultaneous disturbances.	Well-suited for complex events, especially those involving time-varying characteristics.	Performs well for a variety of disturbances, but may not handle complex events like voltage spikes or quick transient faults.	Complex event detection can be improved when PCA is combined with machine learning classifiers, but PCA alone struggles.
Computational Efficiency	Computationally efficient, well-suited for embedded systems in microgrids.	Computationally intensive; requires a large number of calculations for real-time analysis.	Can be computationally expensive, especially when multiple scales are used.	SVM models can be resource-intensive, particularly for real-time monitoring in microgrids.	PCA is computationally efficient, but combining it with machine learning classifiers adds complexity.
Robustness to Noise	Robust against noise, especially when dealing with complex disturbance scenarios.	Sensitive to noise, as it may affect frequency domain characteristics.	More robust than FT to noise, but still can struggle with high-noise levels.	SVM can be robust to noise if well-tuned, but may misclassify noisy or unseen data.	PCA’s performance can degrade in noisy environments unless combined with other techniques like denoising.

**Table 4:** Geometric feature extraction and thresholding scheme in Lissajous-Based PQ disturbance Detection.

Feature	Mathematical expression	Physical interpretation	Purpose in PQ detection	Computational complexity
Lissajous Curve	$\:V\left(t\right)=\:{A}_{v}sin\left(\omega\:t\:+\:{\varphi\:}_{v}\right),\:I\left(t\right)=\:{A}_{i}sin\left(\omega\:t\:+\:{\varphi\:}_{i}\right)$	Parametric plot of voltage vs. current showing phase differences and waveform deformation	Generates disturbance-specific geometric signatures	O(N)
Area Enclosed	$\:Area=\frac{1}{2}\lfloor\sum\:_{k=1}^{n}{V}_{K}{I}_{K+1}-{V}_{K+1}{I}_{K}\rfloor$	**–**	**–**	Indicates phase difference, energy distortion
Skewness (γ)	$\:\gamma=\frac{\frac{1}{N}\sum\:_{i=1}^{N}{\left({x}_{i}-\mu\:\right)}^{3}}{{\sigma\:}^{3}}$	Captures asymmetry in geometric distribut**ion**	Detects asymmetrical faults or unbalanced conditions	O(N)
Kurtosis (κ)	$\:k=\frac{\frac{1}{N}\sum\:_{I=1}^{N}{\left({x}_{i}-\mu\:\right)}^{4}}{{\sigma\:}^{4}}$	Measures peakness of the geometric signal	Distinguishes between sharp transients and low-frequency events	O(N)
Adaptive Threshold	$\:{T}_{j}=\:{\mu\:}_{j}+\:\alpha\:{\sigma\:}_{j}$	Data-driven thresholding using statistical parameters	Enhances sensitivity and specificity for each feature	O(1)
Overall Complexity	**–**	**–**	Efficient feature pipeline avoiding transforms/ML	O(N)

**Table 5:** Comparative analysis of PQ detection Techniques.

Method	Handles overlapping events	Real-time capability	Noise resilience	Computational cost	Need for training data	Remarks
Fourier Transform (FT)	✗	✗	✗	Low	✗	Accurate only in steady-state; poor transient performance
Wavelet Transform (WT)	△ (partially)	△ (limited)	△	High	✗	Mother wavelet selection sensitive; struggles with overlapping events
Machine Learning (e.g., SVM, NN)	✓ (with good data)	△	✗	High	✓ (large datasets)	Sensitive to noise and unseen patterns; training-intensive
PMUs (Phasor Units)	✗	△	✓	High (infrastructure)	✗	Good phase monitoring but fails in transients and high-frequency faults
Adaptive Thresholding (generic)	✗	✓	✗	Low	✗	Threshold tuning is sensitive; lacks adaptability to complex events
Proposed Lissajous-Based Method	✓	✓	✓	Low (linear)	✗	Real-time, scalable, training-free, handles complex and overlapping faults

**Table 6:** Feature response to example PQ disturbances (Hypothetical Values).

Disturbance Type	Area (Lissajous)	Skewness (γ)	Kurtosis (κ)	Interpretation
Normal Operation	≈ 0	≈ 0	≈ 3	Sinusoidal waveform, balanced phase
Voltage Sag	Low	Positive	High	Asymmetry due to temporary drop
Voltage Swell	High	Negative	High	Overshoot results in negative skew and sharper curve
Harmonics	Irregular pattern	Varies	Varies	Multiple lobes in Lissajous; kurtosis detects distortion
Interruption	≈ 0	≈ 0	Very Low	No signal leads to collapsed Lissajous curve
Transient	Medium to High	High	Very High	Sharp spikes cause asymmetry and peaky features

The proposed Lissajous pattern-based power quality (PQ) monitoring technique is quantitatively validated through the comprehensive insights presented in Tables 4 and 5, and Table 6. Table 4 outlines the key geometric features—Area, Skewness, and Kurtosis—with clear mathematical definitions and physical significance, highlighting their roles in capturing phase distortion, waveform asymmetry, and sharpness, respectively. These features are computationally efficient, each exhibiting linear time complexity O(N), making them suitable for real-time embedded applications. Table 5 provides a comparative evaluation of the proposed method against conventional techniques such as Fourier Transform, Wavelet Transform, and machine learning-based approaches. It demonstrates superior performance in terms of accuracy, detection speed, noise resilience, and ability to handle overlapping disturbances, all without requiring training data or complex transformations. Table 6 further supports this by showcasing expected feature behavior across common PQ disturbances, with distinct variations in area, skewness, and kurtosis values under events like sags, swells, interruptions, harmonics, and transients. These discriminative patterns affirm the method’s reliability and robustness, positioning it as a highly effective and scalable solution for dynamic PQ monitoring in smart grids and microgrid environments.

## Conclusions

The proposed methodology introduces a significant advancement in power quality monitoring by leveraging the geometric analysis of dynamic Lissajous figures generated from voltage and current signals. Unlike traditional signal processing approaches, this technique uniquely utilizes visually distinct pattern transformations and statistical metrics—centroid shifts, bounding area, eccentricity, and a Similarity Index (SI)—to diagnose a wide range of PQ disturbances with high accuracy. Numerical experiments validate that centroid deviations effectively track the location and intensity of events, with swells showing the highest Y-direction deviation and corresponding Euclidean distance (0.7071), and pure signals maintaining a baseline at (0,0). This strong correlation ensures robust classification even in complex scenarios involving overlapping events or transient faults. The novelty lies in the integration of adaptive thresholding with shape-based visual analytics, offering an intuitive yet analytically rigorous solution that operates in real-time without requiring grid topology data. The computational efficiency of the method (linear complexity, O(N) supports its implementation on low-power embedded systems, making it ideal for decentralized microgrid environments. Overall, this framework enhances situational awareness, operational efficiency, and the fault tolerance of power systems, and holds strong promise for smart grid applications and renewable energy integration, paving the way toward a more intelligent and resilient electrical infrastructure. Despite its advantages, the proposed method has certain limitations that must be acknowledged. One key limitation is its potential sensitivity to noise and signal distortion, especially in cases where voltage and current signals are severely degraded or exhibit low signal-to-noise ratios, which may affect the clarity of the Lissajous patterns and lead to classification errors. Additionally, while the geometric features used for detection are effective for most common disturbances, the method may face challenges in distinguishing highly similar or overlapping events without additional contextual information or hybrid processing. Furthermore, although adaptive thresholding improves flexibility, it may require fine-tuning under different operating conditions, which could limit its plug-and-play deployment across highly diverse microgrid architectures without initial calibration. The impact of this work is significant in real-world power systems, especially with the increasing penetration of distributed energy resources (DERs), electric vehicle (EV) charging stations, and smart appliances in modern grids. The proposed method empowers utilities and industrial operators to achieve real-time, accurate detection and classification of power quality disturbances without relying on complex transformations or machine learning models. Its compatibility with embedded systems enables deployment in edge devices for continuous, low-latency monitoring. This leads to enhanced grid reliability, faster fault localization, reduced downtime, and proactive maintenance planning. Additionally, its effectiveness under dynamic and noisy grid conditions makes it particularly suitable for smart microgrids, renewable integration points, and critical infrastructure—directly contributing to the advancement of intelligent, self-healing, and net-zero energy systems.

## Data Availability

The datasets used and/or analysed during the current study available from the corresponding author on reasonable request.
